# Introducing a prognostic score for successful treatment‐free remission in chronic myeloid leukaemia

**DOI:** 10.1111/bjh.70409

**Published:** 2026-03-08

**Authors:** Simone Claudiani, Silvia Metelli, Afzal Khan, Guy Hannah, Jenny Byrne, Paolo Gallipoli, Simon J. Bulley, Gillian A. Horne, Kate Rothwell, Mhairi Copland, Richard E. Clark, Fiona Fernando, Andrew J. Innes, Jane F. Apperley, Dragana Milojkovic

**Affiliations:** ^1^ Department of Immunology and Inflammation, Faculty of Medicine Centre for Haematology, Imperial College London London UK; ^2^ Department of Haematology Hammersmith Hospital, Imperial College Healthcare NHS Trust London UK; ^3^ Centre of Research in Epidemiology and Statistics (CRESS) University of Paris, INSERM, INRA Paris France; ^4^ Assistance Publique—Hôpitaux de Paris (APHP) Paris France; ^5^ Department of Haematology King's College Hospital, King's College Hospital NHS Foundation Trust London UK; ^6^ Centre for Clinical Haematology Nottingham University Hospitals NHS Trust Nottingham UK; ^7^ Wellcome‐MRC Cambridge Stem Cell Institute Cambridge UK; ^8^ Department of Haematology Addenbrooke's Hospital, Cambridge University Hospitals, NHS Foundation Trust Cambridge UK; ^9^ Addenbrookes Hospital Cambridge UK; ^10^ Centre for Haematology Barts Cancer Institute, Queen Mary University of London London UK; ^11^ Barts Health NHS Trust Barts UK; ^12^ Department of Haematology St James's University Hospital, Leeds Teaching Hospital NHS Trust Leeds UK; ^13^ Paul O'Gorman Leukaemia Research Centre, School of Cancer Sciences College of Medical, Veterinary and Life Sciences, University of Glasgow Glasgow UK; ^14^ Department of Molecular & Clinical Cancer Medicine University of Liverpool Liverpool UK

**Keywords:** DMR, prognostic score, TFR, TKI

## Abstract

The modern management of chronic myeloid leukaemia (CML) identifies a new therapeutic goal of treatment‐free remission (TFR). Half of CML patients in durable deep molecular response (DMR) (MR^4^ or better) can remain off tyrosine kinase inhibitors (TKIs) without experiencing loss of major molecular response. Despite a large number of TFR studies to date, there are no consistent predictors of successful TFR. We conducted a single‐centre cohort study on 197 patients discontinuing TKIs in DMR ≥1 year and TKI therapy ≥3 years. After TKI discontinuation, 98 patients (49.7%) lost MR^4^; of these, 90 (91.8%; and 45.7% of the whole cohort) lost major molecular response (MMR or MR^3^) after a median of 3.8 months (1–93.3). The 2‐year probability of TFR (pTFR) was 57.7%. In multivariable analysis, male sex, age at diagnosis >40 years, faster achievement of MR^4^ and longer duration of DMR were the only variables significantly associated with higher pTFR. Based on the multivariable analysis results, we built a TFR prognostic score (TPS) able to distinguish three groups with different 2‐year pTFR: good (89.9%), intermediate (61.2%) and poor (18.4%) TFR probability (*p* < 0.0001). We validated the TPS on an independent cohort of 91 patients. We propose that the TPS could become a useful guide for CML clinicians.

## INTRODUCTION

Treatment‐free remission (TFR) is now a realistic goal of treatment for patients with chronic myeloid leukaemia (CML). Approximately 50% of patients who discontinue tyrosine kinase inhibitors (TKIs) after achieving deep molecular response (DMR), defined as a four or more‐log reduction in *BCR::ABL1* (≥MR^4^), are able to remain off treatment indefinitely without losing major molecular response (MMR or MR^3^). Data from the largest TKI stopping trial, EURO‐SKI, showed that the most important variables associated with prolonged TFR were the duration of both TKI therapy and DMR.^1^ The final analysis of this study also showed that blast count at diagnosis, time to MR^4^ and transcript type impact TFR success.^2^

Other clinical trials and retrospective cohort studies confirmed some of the above predictive variables and/or identified other factors.^3^, ^4^, ^5^, ^6^, ^7^, ^8^, ^9^ Differences in eligibility criteria for treatment discontinuation and definitions of relapse have made it difficult to determine optimal candidates for stopping therapy. Recent guidelines provide recommendations for safe clinical practice but have not attempted to predict the likelihood of TFR.^10^, ^11^, ^12^, ^13^

Using a cohort of patients who discontinued therapy at our institution, we developed a prognostic score for successful TFR, validated on an additional cohort of patients treated in other UK centres. We suggest that this TFR prognostic score (TPS) might prove to be a useful clinical tool to advise clinicians and patients considering TFR.

## METHODS

We performed a retrospective analysis of clinical data from patients with CML in whom treatment was discontinued at our centre between 15 March 2009 and 20 November 2023. This constituted the training set (TS). Data cut‐off for analysis was 1 July 2025. The study was approved by our internal review board and all patients gave informed consent. Inclusion criteria were diagnosis of CML in chronic phase, a minimum duration of treatment with TKI of 3 years and discontinuation of TKI after achievement of confirmed ≥MR^4^, durable for at least 1 year. Exclusion criteria were limited clinical information, prior haematopoietic stem cell transplant and TKI resumption before MR^3^ loss. Only the first TKI discontinuation was considered.

TKI failure was defined as per the European LeukemiaNet 2020 criteria.^14^ Duration of MR^4^ before TKI discontinuation was calculated from the date of achievement of ≥MR^4^ sustained until stopping. The times to MR^3^ and MR^4^ were defined as the time between the start of first‐line TKI and the date of the first of at least two consecutive *BCR::ABL1/ABL1* reverse transcription quantitative polymerase chain reaction (RT‐qPCR) ratios ≤0.1% and ≤0.01% on International Scale respectively.

Variables with a *p*‐value ≤0.1 in univariable analysis were used as a pre‐screening step, and subsequently entered into a multivariable Cox proportional hazards model to identify the most influential predictors of the probability of TFR (pTFR) in maintained MR^3^. Measures assessing calibration (calibration slope) and discrimination (*C*‐index) were calculated.^15^, ^16^, ^17^ Loss of MR^3^ while off TKI was the event of interest for deriving pTFR.

A TPS was built from the linear combination of the predictors identified by the Cox model. The TPS was validated on a validation set (VS) which included CML patients who discontinued their TKI at six different UK hospitals and additional patients from our institution who attempted TFR after development of the TS.

Additional section on Methods is available in the Supporting Information.

## RESULTS

### Training cohort

The TS included 197 patients (Table 1). The median age at diagnosis was 46.3 years (16–86), and females constituted the majority of patients (105, 53.3%). The first‐line TKI was imatinib in 167 patients (84.8%), which was also the TKI at discontinuation in 82 (41.6%) patients. Sixteen (8.1%) patients had been enrolled in the DESTINY study.^18^

**TABLE 1:** Baseline patients' characteristics (training and validation cohorts).

Variable	Training set (TS)	Validation set (VS)	*p*‐value
	*N* = 197 (%)	*N* = 91 (%)	
Age at diagnosis, median (range)	46.3 (16–86)	48.3 (19–86)	0.34
Age at stopping, median (range)	58.1 (24–92)	60.6 (26–90)	0.47
Female sex	105 (53.3)	39 (42.9)	0.099
*BCR::ABL1* transcript type			
E14a2	98 (49.7)	33 (36.3)	0.64*
E13a2	67 (34)	18 (19.8)	
E14a2‐E13a2	27 (13.7)	6 (6.6)	
Not available/or other transcript type	4/1 (2.5)	33/1 (37.4)	
Sokal score			
Low or intermediate	98 (49.8)	29 (31.9)	0.14**
High	29 (14.7)	15 (16.5)	
Not available	70 (35.5)	47 (51.6)	
First‐line TKI			
Imatinib	167 (84.8)	74 (81.3)	0.39
Nilotinib	14 (7.1)	7 (7.7)	
Dasatinib	13 (6.6)	10 (11)	
Bosutinib	3 (1.5)	0	
Previous TKI failure	59 (29.9)	16 (17.6)	***0.03***
TKI at stopping			
Imatinib	82 (41.6)	60 (65.9)	***<0.0001*** **(*Imatinib* versus *Others*)**
Dasatinib	48 (24.4)	14 (15.4)	
Nilotinib	51 (25.9)	12 (13.2)	
Bosutinib	9 (4.6)	3 (3.3)	
Ponatinib	3 (1.5)	1 (1.1)	
Asciminib	4 (2)	1 (1.1)	
TKI line at stopping			
1st	102 (51.8)	67 (73.6)	***<0.001***
≥2nd	95 (48.2)	24 (26.4)	
Time to first MR^3^, median in months (range)	11.3 (1.4–102)	12.1 (2.8–124.2)	0.14
Time to first MR^4^, median in years (range)	2.2 (0.3–14.3)	2.9 (0.2–13.9)	0.38
Duration of TKI therapy, median in years (range)	9.5 (3–22.2)	10.3 (3–21.5)	0.65
Duration of MR^4^, median in years (range)	5.9 (1–18.6)	5.5 (1.2–18.3)	0.57
Dose of TKI at stopping			
Standard	29 (14.7)	26 (28.6)	***0.005***
Lower than standard	168 (85.3)	65 (71.4)	
Depth of MR at stopping			
MR^4^	40 (20.3)	21 (23.1)	0.59
≥MR^4.5^	157 (79.7)	70 (76.9)	
Reason for stopping			
DESTINY	16 (8.1)	25 (27.5)	***<0.0001*** **(*DESTINY* versus *others*)**
Guideline/real‐world practice	181 (91.9)	66 (72.5)	
Grade 1 intolerance	65	24	
Grade 2–3 intolerance	46	5	
Pregnancy	10	3	
Patient's request	56	32	
Other	4	2	

*Note*: *p*‐values indicating statistical significance are shown in bold‐italic.

Abbreviations: MR, molecular response; TKI, tyrosine kinase inhibitor.

* The Pearson chi‐squared test was used to compare the distribution of patients with the e14a2 or e14a2‐e13a2 transcript versus those with the e13a2 transcript between the training and validation cohorts; the subjects without available transcript information/with other transcript type were excluded from the analysis.

** Pearson chi‐squared; the subjects without available Sokal score were excluded from the analysis.

The median durations of MR^4^ and TKI therapy before discontinuation were 5.9 years (1–18.6) and 9.5 years (3–22.2) respectively. A history of prior TKI resistance was present in 59 patients (29.9%). At the time of stopping, 102 (51.8%) and 95 (48.2%) patients were receiving first‐line and second‐line or later‐line therapy, respectively, and most patients (*n* = 157, 79.7%) were in MR^4.5^ or deeper molecular response (MR), sustained for a median of 5.2 years (0.25–18.5).

Ninety‐eight patients (49.7%) lost MR^4^, at a median of 3.3 months (0.5–64.3). Of these, 90 (91.8%, and 45.7% of the whole TS) lost MR^3^ after a median of 3.8 months (1–93.3) from TKI interruption and 1.1 months (0–42.4) from MR^4^ loss.

The 2‐year pTFR was 57.7% (95% confidence interval [CI]: 50.8%, 64.4%; Figure S1a) and the median follow‐up in TFR is 73.9 months (6–193). The cumulative 2‐year probability of losing MR^3^ after loss of MR^4^ was 90.5% (95% CI: 82.3%, 95.1%), and was slightly higher for females (94.9% [95% CI: 85.9%, 98.3%]) than for males (82.7% [95% CI: 66.8%, 91.9%]) (*p* = 0.05) (Figure S2a,b).

All 90 patients experiencing MR^3^ loss resumed TKI therapy. Among them, one patient progressed to B‐lymphoid blast crisis after 64 months in TFR and two developed Philadelphia (Ph)‐negative acute myeloid leukaemia at 1.5 and 5 years, respectively, from their MR^3^ loss and died. Further detail about the status at last follow‐up of the whole patient cohort is provided in the Supporting Information—Results and Table S1.

The variables assessed for impact on the pTFR were: *age at diagnosis* (as continuous and dichotomised at different age cut‐offs as previously reported^8^, ^9^, ^19^; Supporting Information—Results; Table S2), *sex*, *Sokal score* (low or intermediate versus high), *transcript type* (e14a2 or e14a2/e13a2 versus e13a2), time to first MR^3^ (*time to MR* ^*3*^) and time to first MR^4^ (*time to MR* ^*4*^) as continuous variables, time to first MR^4^ dichotomised at the median value, *previous TKI failure*, duration of TKI therapy before stopping (*duration of TKI therapy*), first‐generation versus other *TKI first‐line* and *TKI at treatment stop*, duration of MR^4^ or deeper response before stopping (*duration of MR* ^*4*^), enrolment into DESTINY trial,^18^ *TKI dose* (standard versus low dose) and depth of *MR at stopping* (MR^4^ versus MR^4.5^ or deeper).

In univariable analysis (Table 2), the variables most significantly associated with higher pTFR were longer *duration of MR* ^*4*^ (hazard ratio [HR] 0.79 [0.73, 0.85], *p* < 0.0001), absence of *previous TKI failure* (HR 0.54 [0.35, 0.83], *p* = 0.005), shorter *time to MR* ^*3*^ (HR 0.99 [0.98, 1], *p* = 0.01) and *time to MR* ^*4*^ (HR 0.89 [0.83, 0.96], *p* = 0.003), time to first MR^4^ ≤ median (2.2 years) (HR 0.59 [0.38, 0.93], *p* = 0.022), *age at diagnosis* >40 years (HR 0.5 [0.33, 0.76], *p* = 0.001) (Figure S3), male *sex* (HR 0.52 [0.34–0.8], *p* = 0.003) (Figure S4), ≥MR^4.5^ at stopping (HR 0.35 [0.22, 0.55], *p* < 0.0001), longer *duration of TKI therapy* (HR 0.95 [0.9, 0.99], *p* = 0.038), imatinib at stopping (HR 0.55 [0.35, 0.86], *p* = 0.008) and low‐dose TKI at stopping (HR 0.48 [0.29, 0.8], *p* = 0.004).

**TABLE 2:** Univariable and multivariable analyses of variables associated with pTFR.

Variable	*N*	Univariable analysis		Multivariable analysis		
		HR (95% CI)	*p*‐value	HR (95% CI)	*β* coeff.	*p*‐value
Duration of MR^4^ (years)	197	0.79 (0.73–0.85)	*<0.0001*	**0.8 (0.74, 0.87)**	**−0.22**	***<0.0001***
Sex						
Female	105	Reference group	*0.003*	**0.5 (0.32, 0.81)**	**−0.68**	***0.005***
Male	92	0.52 (0.34–0.8)				
Age at diagnosis						
≤40	70	Reference group	*0.001*	**0.54 (0.35, 0.84)**	**−0.61**	***0.006***
>40	127	0.5 (0.33–0.76)				
Age at diagnosis (years)	197	0.99 (0.97–1)	0.237			NE
Previous TKI failure						
No	138	Reference group	*0.005*			NE
Yes	59	1.84 (1.2–2.82)				
MR at stopping						
MR^4^	40	Reference group	*<0.0001*			NS
MR^4.5^	157	0.35 (0.22–0.55)				
Time to first MR^4^ (years)	176	1.12 (1.04–1.2)	*0.003*	**1.14 (1.06, 1.23)**	**0.13**	***0.001***
TKI at stopping						
Imatinib	82	Reference group	*0.008*			NS
2/3GTKI	115	1.81 (1.16–2.82)				
Time to first MR^3^ (months)	174	1.01 (1–1.02)	*0.01*			NE
Duration of TKI therapy (years)	197	0.95 (0.9–0.99)	*0.038*			NE
Time to first MR^4^						
>Median (2.2 years)	89	Reference group	*0.022*			NE
≤Median (2.2 years)	87	0.59 (0.38–0.93)				
TKI dose at stopping						
Low	168	Reference group	*0.004*			NS
Standard	29	2.06 (1.25–3.4)				
Transcript type						
e14a2 or e14a2‐e13a2	125	Reference group	0.065			NS
e13a2	67	1.49 (0.97–2.28)				
DESTINY						
No	181	Reference group	0.52			NE
Yes	16	0.78 (0.36–1.68)				
TKI first line						
Imatinib	167	Reference group	0.75			NE
2GTKI	30	0.91 (0.5–1.64)				
Sokal score						
Low or intermediate	98	Reference group	0.84			NE
High	29	1.06 (0.57–1.98)				

*Note*: *β* coeff., *β* coefficient from the final parsimonious multivariable Cox model. Results of this model are highlighted in bold in the last three columns. NS, not statistically significant; variables marked as NS were evaluated in the multivariable analysis but were not retained in the final parsimonious Cox model. *p*‐values indicating statistical significance are shown in italic for the univariable analysis and in bold‐italic for the final parsimonious multivariable model.

Abbreviations: 2/3GTKI, second‐ or third‐generation TKI; 2GTKI, second‐generation TKI; CI, confidence interval; HR, hazard ratio; MR, molecular response; NE, not entered multivariable analysis; pTFR, probability of TFR; TFR, treatment‐free remission; TKI, tyrosine kinase inhibitor.

The blast count at diagnosis was only available for 127 patients and was significantly associated with pTFR in univariable analysis. In two separate multivariable models, both including this variable and run without multiple imputation due to more than 30% missing data, it did not emerge as an independent predictor (see Tables S3 and S4).

*Transcript type* (e14a2 or e14a2/e13a2) showed a trend towards association with a higher pTFR (HR 0.67 [0.44, 1.03], *p* = 0.065) and entered multivariable analysis.

*Duration of TKI therapy* was not included in the model because of collinearity with *duration of MR* ^*4*^; for the same reason, of *time to MR* ^*4*^ and *time to MR* ^*3*^ only the former was included. Furthermore, as expected, we found a close association between *previous TKI failure* and *time to MR* ^*4*^ (median time to MR^4^ was significantly longer in resistant patients than in optimal responders: 4.8 vs. 1.6 years, respectively, *p* < 0.0001). In view of this, we decided to include in the multivariable analysis only *time to MR* ^*4*^ as a continuous variable over the categorical variable.

The variables that were significant in the multivariable analysis were *duration of MR* ^*4*^ (HR = 0.8 [0.74, 0.87], *p* < 0.0001); *age at diagnosis* >40 years (HR = 0.54 [0.35, 0.84], *p* = 0.006); shorter *time to MR* ^*4*^ (HR = 0.88 [0.81, 0.94], *p* = 0.001); and male *sex* (HR = 0.5 [0.32, 0.81], *p* = 0.005). The model calibration slope was 0.98 and the optimism‐corrected *C*‐index 0.748 (95% CI: [0.70, 0.79]).

We evaluated two alternative multivariable models: the first replaced *time to MR* ^*4*^ with *previous TKI failure*; and the second replaced both *time to MR* ^*4*^ and *duration of MR* ^*4*^ with *previous TKI failure* and *duration of TKI therapy* respectively. In the latter model, *transcript type* emerged as a significant factor. However, both alternative models showed inferior performance based on model metrics (see Tables S5 and S6).

### Building the TPS

We constructed the TPS using the four predictors that were significantly associated with TFR in the multivariable analysis. The linear predictor (prognostic index [PI]) of the final Cox model is the quantity used to derive our proposed TPS:

$$\lambda \left(t\right)=\lambda_{0}\left(t\right)\cdot \exp(\mathrm{PI}),$$

$$\begin{array}{c} \lambda \left(t\right)=\lambda_{0}\left(t\right)\cdot \exp\left(\beta_{1}\cdot \mathrm{Age}>40+\beta_{2}\cdot \mathrm{Sex}\;\text{Male}+\beta_{3}\cdot \right.\\ \;\left.\text{Duration}{\mathrm{MR}}^{4}+\beta_{4}\cdot \text{TimeTo}{\mathrm{MR}}^{4}\right) \end{array}$$

where $\lambda_{0}\left(t\right)$ is the baseline hazard function and $\left(\beta_{1},\beta_{2},\beta_{3},\beta_{4}\right)$ are the regression coefficients of the model (Table 2).

We centred the TPS on ‘average risk’ by subtracting the training cohort PI mean value (*μ*_PI_ = −1.75).

$$\begin{array}{c} \mathrm{TPS}=\left(-0.61\cdot \mathrm{Age}>40-0.68\cdot \mathrm{Sex}\;\text{Male}-0.22\cdot \right.\\ \;\left.{\text{DurationMR}}^{4}+0.13\cdot {\text{TimeToMR}}^{4}\right)-\left(-1.75\right) \end{array}$$

**FIGURE 1:**
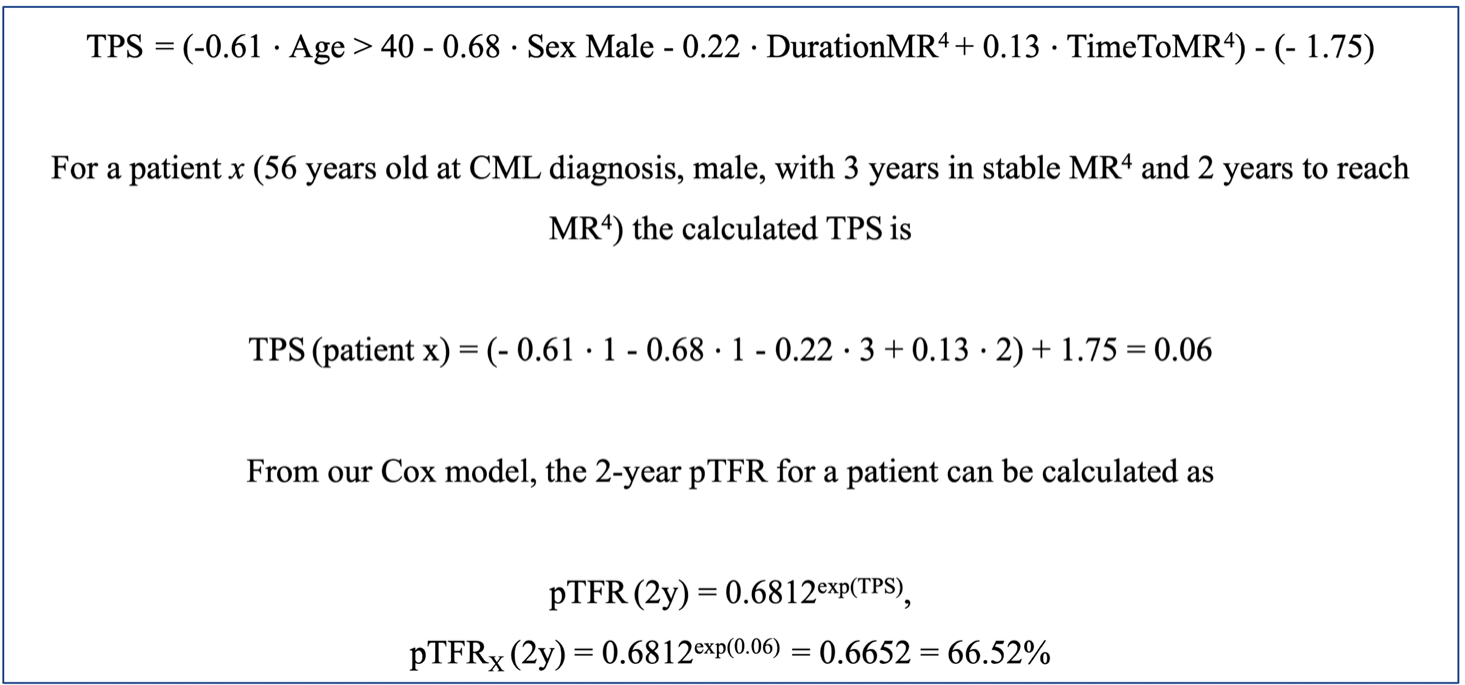
How to calculate the TFR prognostic score (TPS). TPS = TFR prognostic score; 0.6812 is the estimate for the baseline survival function at 2 years for pTFR. The estimates for the baseline survival function at 1 and 3 years for pTFR are 0.7086 and 0.6668 respectively. The patient in the example above would fit into the intermediate outcome group (given his calculated TPS of 0.06; refer to Table 3). pTFR, probability of TFR; TFR, treatment‐free remission.

**FIGURE 2:**
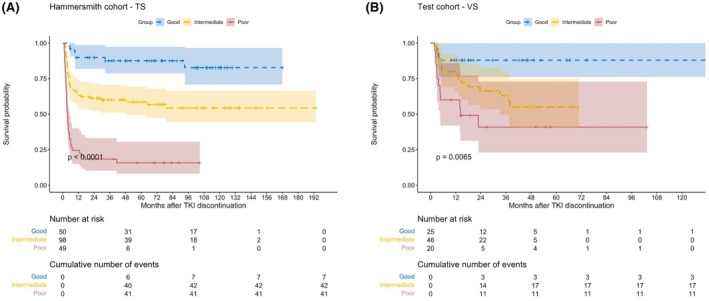
Probability of TFR (pTFR) in sustained MR^3^ for the training set (TS) and the validation set (VS). The figure illustrates the three groups of patients identified by the TFR prognostic score (TPS) for both TS (A) and VS (B), with significantly different probabilities of maintained MR^3^ (pTFR) after TKI discontinuation. TFR, treatment‐free remission; TKI, tyrosine kinase inhibitor.

The variables are categorized as: Age=0 if ≤40 years and 1 otherwise, Sex=1 if male and 0 if female, while Duration of MR^4^ and Time to MR^4^ are both continuous (in years). For instructions on calculating the TPS for an individual patient (see Figure 1 and the Microsoft Excel calculator included in the Supporting Information). Three patient groups with good (*n* = 50), intermediate (*n* = 98) and poor (*n* = 49) TFR probability were obtained from the TPS by placing cut‐off points at meaningful quantiles (Table 3; Figure S5). The corresponding 2‐year pTFR values were 89.9% (95% CI: [81.8%, 98.7%]), 61.2% (95% CI: [52.3%, 71.6%]) and 18.4% (95% CI: [10.2%, 33.1%]) respectively (overall log‐rank test *p* < 0.0001) (Figure 2A).

**TABLE 3:** TPS values for the three different pTFR outcome groups.

pTFR outcome group	TPS
Good	TPS <−0.68065
Intermediate	−0.68065 ≤ TPS < 0.77448
Poor	TPS ≥ 0.77448

Abbreviations: pTFR, probability of TFR; TPS, treatment‐free remission prognostic score.

### Validation cohort

The TPS was tested on 91 patients (Table 1). This group was significantly different from the TS in that a higher proportion of patients discontinued imatinib (65.9% vs. 41.6%) and their first‐line TKI (73.6% vs. 51.8%), a higher percentage of patients were on standard‐TKI dose at stopping (28.6% vs. 14.7%), a greater number of patients were enrolled into DESTINY trial (25 vs. 16) and a lower proportion had TKI resistance (17.6% vs. 29.9%). The distribution of patients by other clinical variables was comparable between the two cohorts.

Thirty‐nine (42.8%) patients lost MR^4^ at a median of 3 months (1.2–23.6) from TKI interruption. Of these, 31 patients (79.5%, and 34% of the whole VS) lost MR^3^, at a median of 4.6 months (1.87–37.8) after stopping TKI and 1.48 months (0–29.6) from MR^4^ loss.

The 2‐year pTFR was 66.6% (95% CI: 55.7%, 76%) and the median follow‐up of patients in TFR was 35.8 months (3–132) (Figure S1b).

The cumulative 2‐year probability of losing MR^3^ after MR^4^ was 81% (95% CI: 64%, 91.1%) and, similarly to the TS, it was higher for females (100%) than for males (65.9% [95% CI: 42.6%, 83.4%]) (*p* = 0.003) (Figure 2c,d).

In this population, the TPS was applied to a total of 91 patients for pTFR and identified 25, 46 and 20 patients in the good, intermediate and poor outcome groups respectively. The 2‐year pTFR were 88% (95% CI: [76.1%, 100%]), 66.4% (95% CI: [53.3%, 82.7%]) and 40.9% (95% CI: [22.9%, 72.9%]) respectively (overall log‐rank test *p* = 0.006) (Figure 2B).

The model calibration was satisfactory, with a calibration slope of 0.58 and a *C*‐index of 0.65 (95% CI: [0.55, 0.74]).

## DISCUSSION

We showed four variables to be associated with a higher pTFR, namely age at diagnosis >40, male sex, faster achievement and longer duration of DMR, and built a statistical model to separate patients into good, intermediate and poor outcome groups.

The model was validated on an independent cohort, despite differences in the characteristics between this cohort and the TS. The most relevant difference was the shorter TFR follow‐up in the validation cohort, which was reflected in the distinct overall pTFR observed between the two groups.

We observed that the strongest factor impacting on the achievement of TFR is the duration of MR^4^ which confirms the finding of the EURO‐SKI study.^1^ In addition, we found that TKI duration was significantly associated with higher TFR rates, as previously shown.^1^, ^3^, ^4^, ^18^ In the final analysis from EURO‐SKI,^2^ TKI duration emerged as a stronger prognostic factor than the duration of DMR, as, along with transcript type and blast count at diagnosis, it remained significantly associated with sustained MMR during the late relapse phase (i.e. between six and 36 months after treatment discontinuation).

Conversely, in our study, *duration of MR4* was more strongly associated with higher pTFR and, for this reason, and for its collinearity with *duration of TKI therapy*, it was chosen for our final TPS model.

An additional finding from our study is that the loss of MR^4^ is almost inevitably followed by loss of MR^3^ and this raises the possibility of changing the criterion for re‐starting therapy. Of note, lack of MR^4^ at 36 months after discontinuation of TKI was highly predictive of subsequent loss of MMR in the AFTER‐SKI study.^20^

The impact of age at diagnosis on the risk of dying of a CML‐related cause requires elucidation.^21^, ^22^ The favourable impact of older age at diagnosis in TFR was originally reported in the ISAV study^8^, ^19^ and later confirmed by our previous report and other studies.^9^, ^23^, ^24^, ^25^ In our analysis, age at diagnosis had its strongest impact on TFR when analysed as a dichotomous variable, with patients aged 40 years or younger showing lower probabilities of TFR. When age was modelled as a continuous variable, no significant association with TFR was observed, suggesting a non‐linear effect better captured by a threshold. The biological explanation for this is unclear: aged haematopoietic stem cells have a lower regenerative potential and slower cell cycle progression^26^ but the effect of age on Ph‐positive cells is unknown.

Our finding that age ≤40 years may be associated with a lower likelihood of TFR remains relevant in principle also to patients from developing countries, where the median age at CML diagnosis is substantially lower,^27^ as this is likely due to the generally younger age structure of these regions.^28^ Importantly, younger CML patients should not be discouraged from pursuing TFR, as other determinants, among which the duration of DMR appears to play the most prominent role, also influence TFR success. Nevertheless, further studies are warranted to assess whether different disease biology contributes to the younger median age in these regions.

Prior TKI failure has been reported to be associated with an increased risk of relapse in trials of discontinuation,^6^, ^7^ and is a relative contraindication to stopping in the European and British guidelines,^10^, ^11^, ^13^ but not in the American recommendations.^12^ The negative impact of TKI resistance on pTFR may partly explain why a shorter time to first MR^4^ was associated with higher TFR probability. We found a strong association between these two clinical variables and chose the latter, as a continuous variable, to build our model. The predictive value of early response for successful TFR has been described by the Australian group reporting that shorter *BCR::ABL1* transcript halving‐times in the first three months of TKI therapy predict a higher likelihood of subsequent TFR.^29^ Furthermore, in the final EURO‐SKI analysis, time to DMR was included in one of the three significant multiple logistic regression models identified.^2^

In real‐world practice, many patients achieve durable DMR after prior TKI resistance and express an interest in stopping treatment. Their inclusion in our study created a model that can advise on the appropriateness of a trial of discontinuation. We have previously shown that TFR is possible in non‐optimal responders,^30^ and the adverse impact of TKI resistance may be abrogated by prolonged time in MR^4^.

A positive influence of male sex on TFR was originally shown by STIM trial^3^ but has not been subsequently confirmed. We found that male patients had a significantly higher pTFR compared to females. A possible hypothesis is the known sex differences in immune cell composition and immune responses.^31^, ^32^, ^33^

Previously we reported that transcript type was associated with different TFR rates in CML patients,^24^, ^34^ later confirmed by others,^29^, ^35^, ^36^ including the EURO‐SKI study.^2^ In our alternative Cox regression model excluding the DMR variables (*time to MR* ^*4*^ and *duration of MR* ^*4*^), *transcript type* retained statistical significance along with *age at diagnosis*, *sex*, *duration of TKI therapy* and *previous TKI failure*. This suggests that transcript type may play an important role in TFR and remain a strong TFR predictor when DMR‐related variables are not considered. Interestingly, in the latest EURO‐SKI analysis, transcript type emerged as significantly associated with the probability of maintaining MMR 36 months after TKI stop only in the multiple logistic regression model excluding DMR‐related variables.^2^

Finally, we report a case of lymphoid blast crisis occurring during the fifth year of TFR, which underscores the importance of continued clinical and molecular surveillance, even in long‐term TFR.^37^

The main limitation of our study is the relatively low patient numbers. To address sample size issues, we adopted shrinkage and over‐optimism correction, although residual confounding cannot be excluded. Future larger validation sets would be desirable to provide more precise calibration estimates.^38^ Although similar TFR rates have been described in patients receiving first‐ or second‐generation TKIs^6^ as first‐line therapy, it is important to note that the majority of patients in both the TS and VS were treated with imatinib. Due to limited information, the role of peripheral blast count at diagnosis in the whole training cohort was not evaluable.

We believe that the TPS serves as a flexible backbone that can be applied in independent, larger patient cohorts and potentially enhanced by the incorporation of new variables. If validated in such cohorts, this prognostic score may allow for a more precise and individualized assessment of TKI discontinuation.

## AUTHOR CONTRIBUTIONS

S.C. designed the study, collected and analysed the data, interpreted the results and wrote the manuscript; S.M. analysed the data and wrote the manuscript; A.K., G.H., J.B., P.G., S.J.B., G.A.H, K.R., M.C. and R.E.C. collected the data and reviewed the manuscript; F.F. and A.J.I. reviewed the manuscript; J.F.A. and D.M. wrote the manuscript.

## CONFLICT OF INTEREST STATEMENT

S.C.: none. S.M.: none. A.K.: none. G.H.: Novartis (speaker's fees, advisory board); Pfizer (speaker's fees); Incyte (speaker's fees); Astellas (advisory). J.B.: Novartis and Incyte (consultancy and speaker fees). P.G.: none. S.J.B.: McKinsey & Company, Taylor & Francis Group, Vertex Pharmaceuticals (consultancy); Hartley Taylor, Janssen, Sanofi, Therakos (honoraria). G.A.H.: Novartis, GSK, Astellas, Jazz Pharmaceuticals and Amgen (honoraria). K.R.: honoraria for speaking engagements or conference attendance from Novartis, Incyte, Pfizer. M.C.: research funding from Cyclacel and Incyte; advisory board member for Novartis, Incyte, Pfizer, Ascentage, Crossbow, Servier; honoraria from Astellas, Jazz Pharmaceuticals, Novartis, Incyte, Pfizer, Servier and Janssen. R.E.C.: none. F.F.: none. A.J.I.: none. J.F.A.: Ascentage, Incyte, Novartis, Paladin and Terns (honoraria); Incyte and Pfizer (research funding). D.M.: Ascentage, Incyte, BMS, Novartis, Pfizer and Terns (honoraria).

## ETHICS STATEMENT

The study was approved by our internal review board and all patients gave informed consent.

## Supporting information


Data S1.

Data S2.

## Data Availability

The data that support the findings of this study are available from the corresponding author upon reasonable request.
