# Supplementary material for: Introducing a prognostic score for successful treatment‐free remission in chronic myeloid leukaemia
Source: Br J Haematol. 2026 Mar 8;208(5):1660–8. doi: 10.1111/bjh.70409 (PMC13176515; doi:10.1111/bjh.70409)
Supplement: Supplementary file 2 — Data S2. [file BJH-208-1660-s002.pdf]

Supporting Information

Introducing a prognostic score for successful treatment-free remission in chronic myeloid leukaemia

Additional section on Methods

Standard response definitions were used[1] and molecular monitoring was performed according to current guidelines[2,3]. Low-dose TKI was defined as any TKI dose lower than the standard recommended TKI doses.

The calibration slope assessed the agreement between predicted and observed risk estimates (with a slope of 1 suggesting perfect calibration, above 1 suggesting underestimation of risks by the model, and below indicating overestimation), while the C-index assessed the accuracy of prediction (with a value of 1 indicating perfect discrimination and a value of 0.5 indicating no discrimination). Optimism-corrected measures and model coefficients shrinkage were used to correct for over-optimism[4,5].

Cox Proportional Hazards model

Proportional hazard assumptions were tested with Schoenfeld residuals[6]. The dataset was divided into two datasets: a training set, used to derive the model, and a test set, used to validate the risk model. Data were assumed to be missing at random, and values for the missing predictors were imputed using multiple imputation techniques based on chained equations (MICE)[7]. A total of 50 imputed datasets were generated. We applied the following rule: variables exceeding 30% missing were excluded from the model. An imputation model was defined for each variable with ≤ 30% missing data.

Because molecular monitoring was performed at predefined intervals, patients without documented loss of MR^3 were censored at the date of their last available molecular assessment. Death occurring during TFR can be considered a competing event, and in principle a competing risk modelling framework (e.g. the Fine and Gray model) could be applied. However, a competing risk analysis for death was not performed, as molecular status at the time of death was unavailable; therefore, it was not possible to determine whether molecular relapse had occurred prior to death. Treating death as a competing event would have required unverifiable assumptions regarding MR^3 status at death. A detailed description of causes of death in the TFR cohort is provided in a separate section below.

To identify three groups, the cut-off points were placed at the 25th and 75th centiles of the TPS in the training set[5]. Unequal group sizes were chosen because they allow for the identification of patients with extreme prognoses. However, with a small sample size, this approach may result in very few patients in one of the three groups. Alternatively, equal-sized groups can be constructed, but this may reduce

the ability to detect extreme patients and make it harder to group very similar patients together. External validation was conducted following principled guidelines[8].

All statistical analyses have been conducted using R statistical software, version 3.5.1, using *survival*, *MASS*, *survminer, Rms* and *shrink* packages.

Additional section on Results

Long-term outcomes after failed TFR attempt in the training cohort

Of the 90 patients who experienced loss of MR^3 following TKI discontinuation, all resumed therapy. Among them, one patient progressed to B-lymphoid blast crisis after 64 months in TFR. Notably, the most recent *BCR::ABL1* assessment, performed four months prior to blast phase, demonstrated a sustained deep molecular response in the absence of TKI therapy (0.002% on the International Scale). The patient had been diagnosed with chronic-phase CML 15.3 years before the onset of blast crisis and exhibited no high-risk features at diagnosis or during prior therapy. He re-achieved deep molecular response after UKALL60+ chemotherapy[9] with dasatinib, remains alive and in DMR nine months from the onset of blast crisis, and is currently receiving consolidation therapy with blinatumomab and imatinib.

Of the remaining 89 patients who lost MR^3, 85 (95.5%) regained MR^3 after a median of 2.5 months (0.5-28) from TKI resumption; of the four patients not re-achieving MR^3, their follow-up after resuming their TKI was 1.2, 7.9, 1.5 and 9.1 months. The latter two subsequently died of uterine adenocarcinoma and metastatic colon adenocarcinoma (at 3.5 and 19.1 months from TKI restart, respectively).

Of the 85 patients regaining in MR^3, two subsequently developed Philadelphia-negative AML at 1.5 and 5 years from their MR^3 loss and died. Among 83 remaining patients, eight died while still maintaining $\geq MR^3$, due to lung adenocarcinoma, glioblastoma, gastric adenocarcinoma, Parkinson's disease, aortic stenosis, colonic adenocarcinoma, or, in two cases, unknown causes. A further 75 patients were alive at last follow-up at a median of 54.7 months from MR^3 loss and their responses were: MR^2 in 3 (4%), MR^3 in 18 (24%) and DMR in 54 (72%), after medians of 47.7 (22.5-53.8), 45.8 (1-134.9) and 56.7 months (3.5-170.3), respectively, from TKI resumption.

Molecular status and subsequent deaths of patients who were in TFR at last available molecular follow-up

Among the 107 patients who did not experience loss of MR^3, molecular response at the last molecular follow-up was MR^3 in two patients (1.9%), MR^4 in 17 patients (15.9%), and $MR^{4.5}$ or deeper in 88 patients (82.2%). Eleven of the 107 patients (10.3%) who remained in ongoing TFR at the last molecular follow-up subsequently died. Molecular status at the time of death was unknown. The table below summarizes their clinical details.

Supplementary Table 1. Subsequent deaths among patients in ongoing TFR at last molecular follow-up.

ID	Sex	Age at death	TFR follow-up in months	Months from last follow-up* until death	Resumed TKI	Cause of death
1	M	85	66.1	64.4	*Unknown*	*Unknown*
5	F	94	26.8	2.2	No	Alzheimer's disease
7	M	90	111	4.1	No	COVID-19
44	M	87	134.5	3.2	No	Pulmonary arterial hypertension
59	M	82	52	12.4	No	Metastatic oesophageal cancer
84	F	74	63.6	13.1	No	Alzheimer's disease
90	F	68	6.1	7.4	No	Cardiac amyloidosis
99	M	88	19	3.9	No	Pneumonia
114	F	78	64.3	45.4	No	Alzheimer's disease
189	M	66	39.1	4.1	No	Progressive CKD
255	M	75	45.5	8.6	No	Progressive CKD

Legend: * date of the last molecular test while off TKI (RT-qPCR for *BCR::ABL1*); CKD: chronic kidney disease.

Exploring the effect of age at diagnosis on the probability of treatment-free remission

When *Age at diagnosis* was included as a continuous variable in univariable Cox regression, it was not significantly associated with maintaining MR^3 after TKI discontinuation (HR 0.99, 95% CI 0.97–1, p = 0.237).

Although continuous variables are generally preferred over dichotomized ones, we chose to explore a potential threshold effect based on accumulating evidence supporting age-specific cut-offs, particularly since the continuous analysis did not demonstrate a clear or linear association. Several candidate *Age at diagnosis* cut-offs — 25, 30, 35, 37.5, 40, 42.5, 45, 50, 55, 60, and 65 years — were evaluated in the training cohort to identify any age thresholds that would better separate Kaplan–Meier curves. Among these, age 40 years produced the greatest separation (lowest log-rank p-value = 0.001) and was therefore selected for subsequent analyses.

To assess the robustness of this finding, the 40-year threshold was also tested in the independent validation cohort; the log-rank p-value was 0.06, but the direction of effect was consistent, suggesting that the observed pattern was not cohort-specific. The multivariable Cox including *Age at diagnosis* as a continuous variable was also fit, and its model's goodness of fit compared to the main model including *Age at diagnosis* as a categorical variable (cut-off at 40 years), which resulted in the former model having a poorer performance (model with age continuous Bayesian information criterion [BIC]: 856.20, main model BIC: 848.5).

The Supplementary Table 2 summarizes the log-rank p-values and univariable Cox hazard ratios for all candidate *Age at diagnosis* cut-offs in both cohorts.

Supplementary Table 2. Log-rank p-values and univariable Cox hazard ratios for candidate *Age at diagnosis* thresholds on the probability of treatment-free remission (pTFR) in the training (TS) and validation (VS) sets.

Age at dx cut-off (years)	N patients below/above cut-off (TS)	Log-rank p-value (TS)	N patients below/above cut-off (VS)	Log-rank p-value (VS)	Cox HR (TS)	95% HR CI (TS)	Cox HR (VS)	95% HR CI (VS)
25	18/179	0.135	4/87	0.682	0.6	0.31-1.17	1.51	0.2-11.1
30	26/171	0.247	10/81	0.238	0.71	0.4-1.26	0.56	0.21-1.47
35	45/152	0.029	18/73	0.61	0.605	0.38-0.95	0.8	0.34-1.87
37.5	52/145	0.029	22/69	0.48	0.616	0.4-0.96	0.76	0.35-1.65
**40**	**70/127**	**0.001**	**27/64**	**0.06**	**0.5**	**0.33-0.76**	**0.5**	**0.25-1**
42.5	83/114	0.11	33/58	0.1	0.715	0.47-1.08	0.56	0.27-1.13
45	92/105	0.716	36/55	0.094	0.926	0.61-1.4	0.55	0.27-1.11
50	120/77	0.936	48/43	0.85	1.01	0.67-1.55	0.933	0.46-1.9
55	141/56	0.735	60/31	0.84	0.923	0.58-1.46	0.925	0.435-1.96
60	163/34	0.476	74/17	0.23	0.813	0.46-1.44	0.532	0.186-1.52
65	179/18	0.51	85/6	0.34	0.77	0.36-1.67	0.38	0.052-2.8

Legend: dx = diagnosis; N = number of; HR = hazard ratio; TS = training set; VS = validation set; CI = confidence interval.

Supplementary Figure 1a. Probability of survival in sustained MR³ after TKI discontinuation (pTFR) in the training cohort.

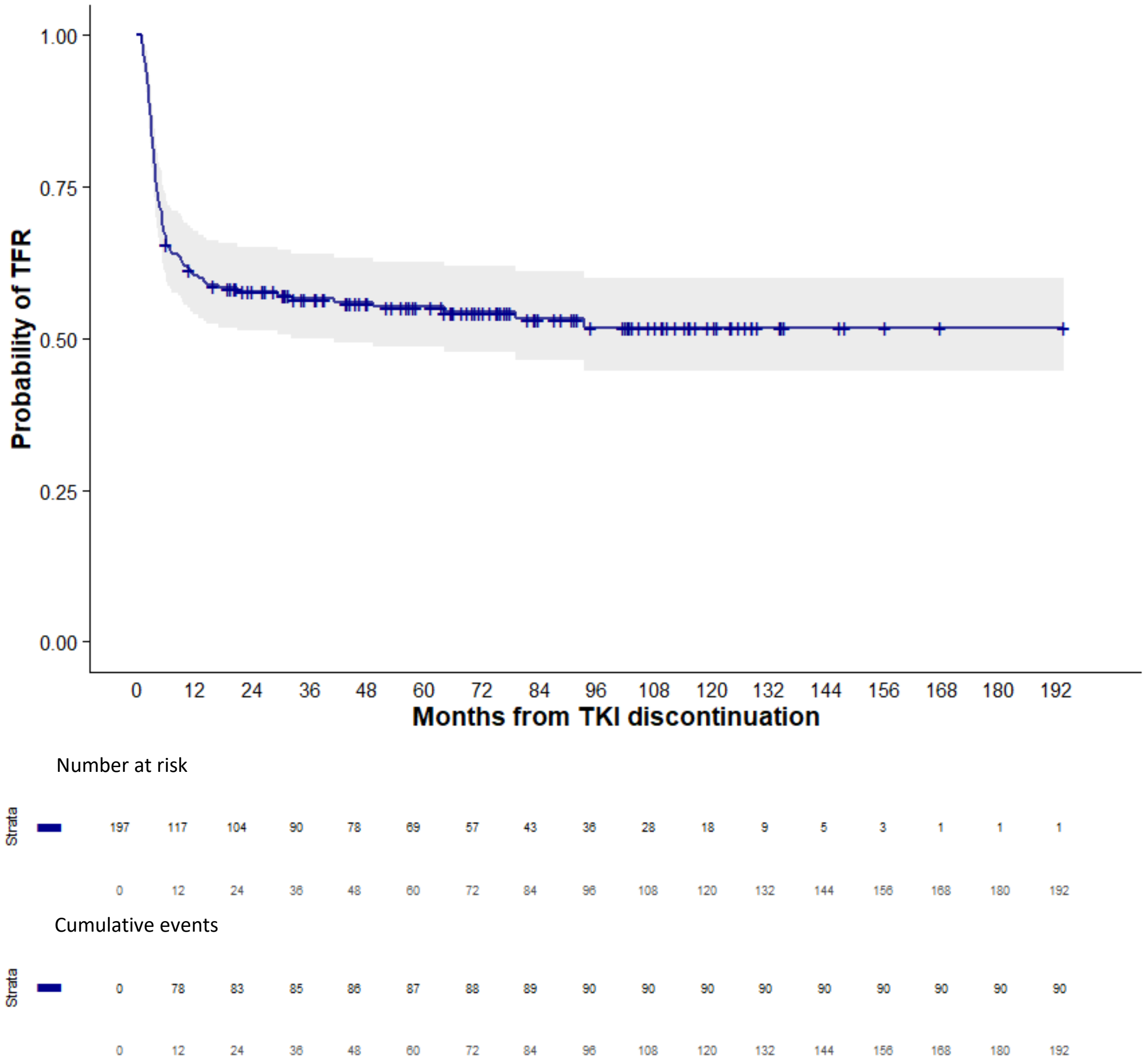

Legend to S. Figure 1a:

Kaplan-Meier curve illustrating the pTFR in the training cohort (n = 197), with grey shaded area representing the 95% confidence interval.

Supplementary Figure 1b. Probability of survival in sustained MR³ after TKI discontinuation (pTFR) in the validation cohort.

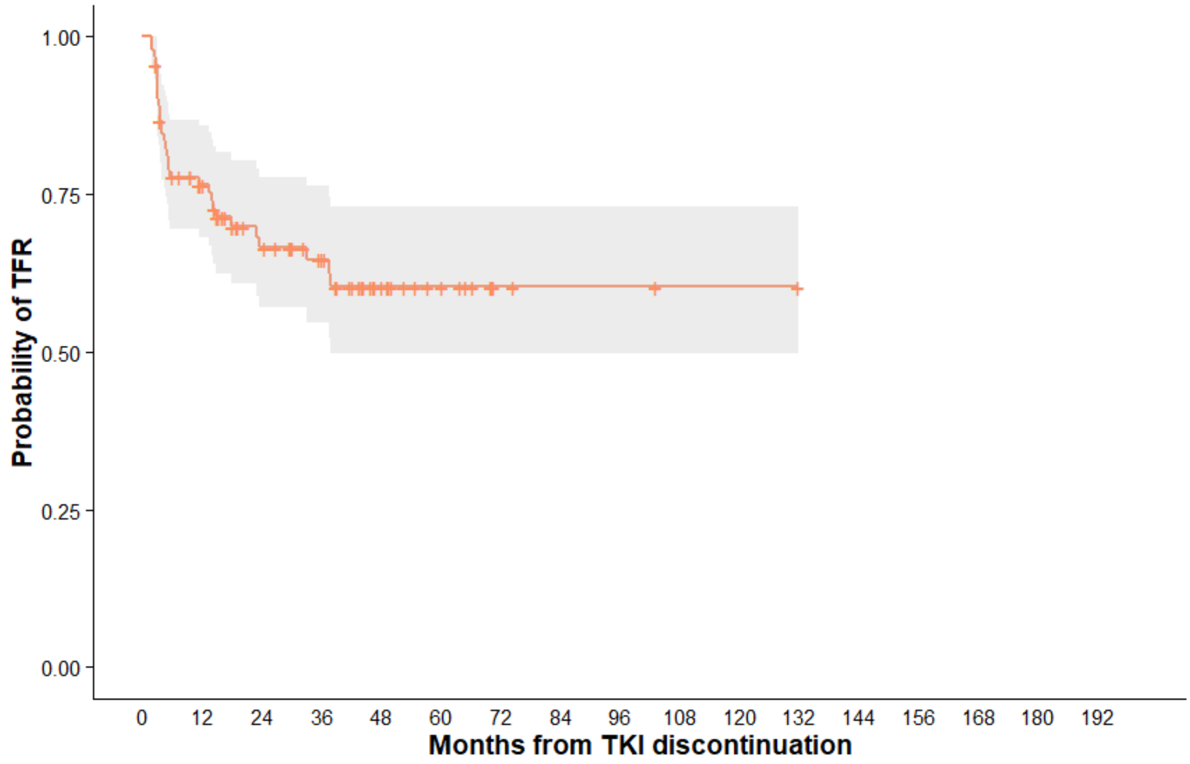

Number at risk

Strata	0	12	24	36	48	60	72	84	96	108	120	132	144	156	168	180	192
	91	61	40	32	17	10	3	2	2	1	1	0	0	0	0	0	0

Cumulative events

Strata	0	12	24	36	48	60	72	84	96	108	120	132	144	156	168	180	192
	0	21	28	29	31	31	31	31	31	31	31	31	31	31	31	31	31

Legend to S. Figure 1b:
Kaplan-Meier curve illustrating the pTFR in the validation cohort (n = 91), with grey shaded area representing the 95% confidence interval.

Supplementary Figure 2: Cumulative probability of MR^3 loss after MR^4 loss following TKI discontinuation in the training and in the validation cohorts.

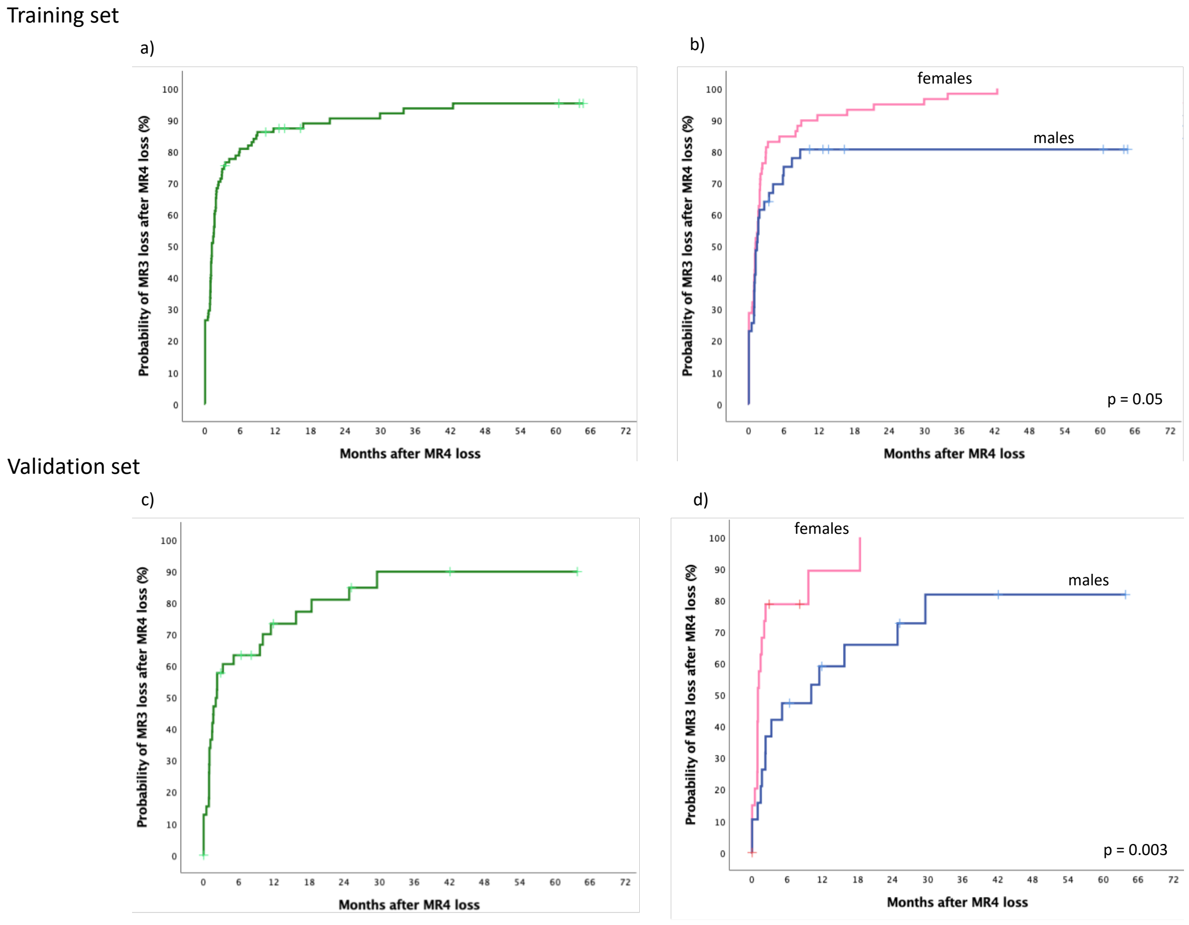

Legend to S. Figure 2:
Kaplan-Meier curves showing the cumulative probability of MR^3 loss following MR^4 loss after TKI discontinuation in the training (a) and validation (c) cohorts, also stratified by sex (b and d, respectively). P-values were calculated using the log-rank test.

Supplementary Figure 3. Probability of survival in sustained MR[3] after TKI discontinuation (pTFR) stratified by *Age at diagnosis*.

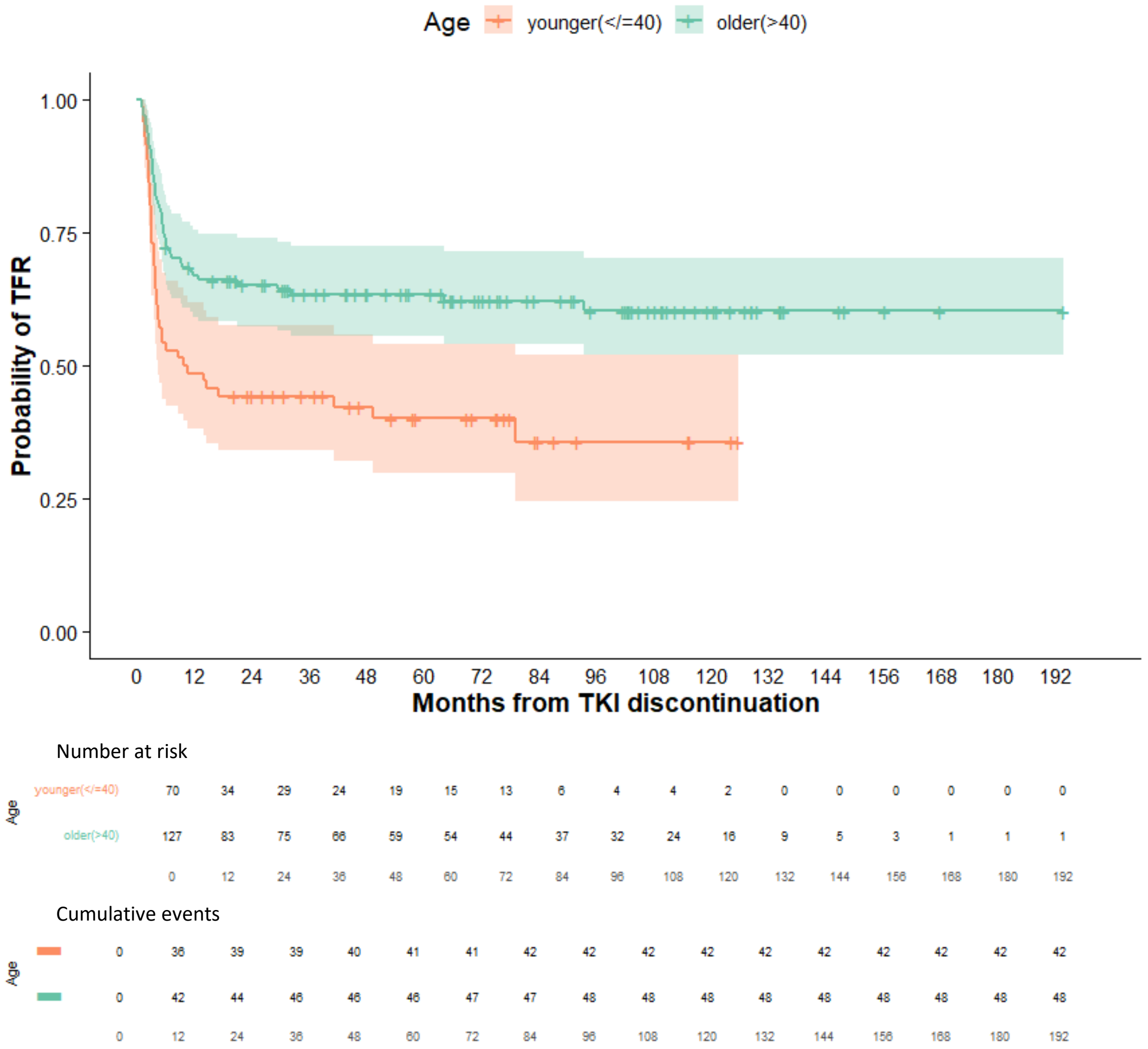

Legend to S. Figure 3:
Kaplan-Meier curve showing the pTFR in the training cohort (n = 197), stratified by *Age at diagnosis* (> 40 years [green line] versus ≤ 40 years [orange line]). Colored shaded areas represent the 95% confidence interval.

Supplementary Figure 4. Probability of survival in sustained MR[3] after TKI discontinuation (pTFR) stratified by *Sex*.

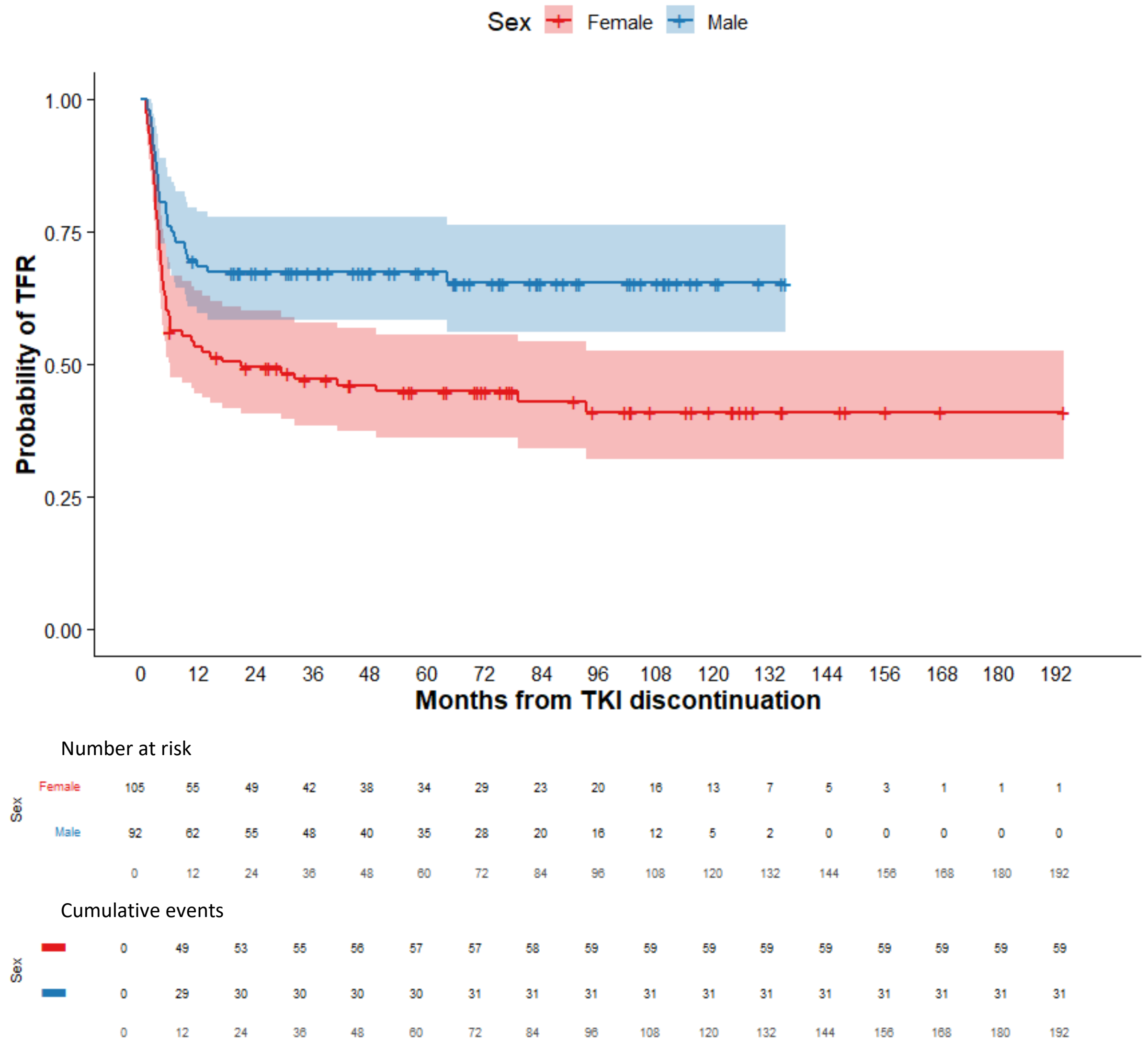

Legend to S. Figure 4:
Kaplan-Meier curve showing the pTFR in the training cohort (n = 197), stratified by *Sex* (male [blue line] versus female [red line]). Colored shaded areas represent the 95% confidence interval.

Supplementary Figure 5. TPS distribution in the training cohort.

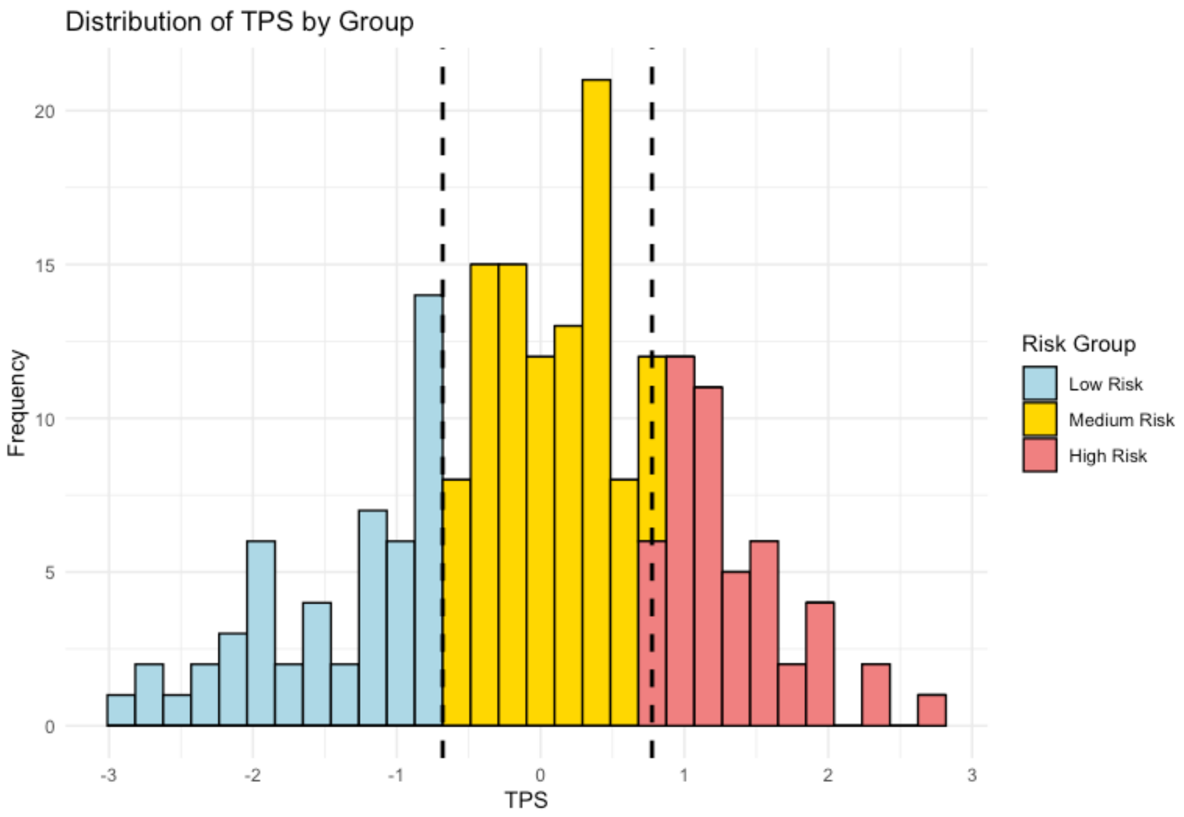

Legend to S. Figure 5: The histogram shows the distribution of TPS in the training cohort (n = 197), categorized into three TFR outcome groups (refer to Table 3 in the main manuscript): low risk (good outcome, n = 50; TPS < -0.68065), intermediate risk (intermediate outcome, n = 98; -0.68065 ≤ TPS < 0.77448), and high risk (poor outcome, n = 49; TPS ≥ 0.77448).

Multivariable Cox regression analysis in the training cohort subgroup with available *blast count at diagnosis*.

In univariable analysis on 127 patients with available *blast count at diagnosis* this variable resulted significantly associated with pTFR: HR 1.1 (95% CI:1.02,1.2), p = 0.015. However, in multivariable analysis, it did not remain significant.

Supplementary Table 3. Training cohort subgroup with available *blast count at diagnosis*: multivariable analysis for pTFR (n = 115) including the variables with p value ≤0.1 in univariable analysis and choosing the DMR-related variables instead of *Previous TKI Failure* and *Duration of TKI therapy*.

Variable	Beta coeffient	P-value	HR	HR (95% CI, lower)	HR (95% CI, upper)
**Sex (Male)**	-0.809	**0.018**	0.445	0.228	0.868
Age at diagnosis (>40 years)	-0.574	0.07	0.563	0.302	1.049
Blast on peripheral blood at diagnosis (%)	0.067	0.216	1.070	0.961	1.190
MR at stop ($MR^{4.5}$ or deeper)	0.081	0.825	1.084	0.530	2.218
TKI dose at stop (lower than standard)	-0.468	0.272	0.626	0.272	1.443
**Time To MR^4 (years)**	0.122	**0.017**	1.129	1.022	1.248
**Duration of MR^4 (years)**	-0.163	**0.003**	0.850	0.762	0.948
Imatinib at stop	-0.312	0.381	0.732	0.364	1.472

Supplementary Table 4. Training cohort subgroup with available *blast count at diagnosis*: multivariable analysis for pTFR (n = 127) including the variables with p value ≤0.1 in univariable analysis and choosing *Previous TKI Failure* and *Duration of TKI therapy* instead of the DMR-related variables.

Variable	Beta coeffient	P-value	HR	HR (95% CI, lower)	HR (95% CI, upper)
**Sex (Male)**	-0.727	**0.015**	0.483	0.269	0.870
Age at diagnosis (>40 years)	-0.416	0.15	0.66	0.374	1.163
Blast on peripheral blood at diagnosis (%)	0.069	0.157	1.071	0.974	1.179
MR at stop ($MR^{4.5}$ or deeper)	-0.449	0.130	0.607	0.318	1.158
TKI dose at stop (lower than standard)	-0.508	0.133	0.602	0.310	1.167
Previous TKI failure (yes)	0.520	0.134	1.682	0.852	3.322
Duration of TKI therapy (years)	-0.029	0.471	0.972	0.898	1.051
Imatinib at stop	-0.518	0.095	0.596	0.325	1.1094

Supplementary Table 5 and 6. Alternative Cox regression models where *Time To MR^4* and *Duration of MR^4* were not included in the multivariable analysis.

Supplementary Table 5. Alternative Model 1 (n = 197): *Time to MR^4* substituted by *Previous TKI failure*. Model's optimism-corrected C-index: 0.73. Model's Bayesian information criterion (BIC): 848.47 [Main model BIC: 848.5].

Variable	Multivariable analysis for pTFR (n=197)	
	HR (95% CI)	P-value
Duration of MR^4 (years)	0.80 (0.74, 0.86)	<0.0001
Previous TKI Failure		
- no (reference group)		
- yes	1.94 (1.25, 3)	0.003
Age at diagnosis		
- ≤40 (reference group)		
- >40	0.57 (0.37, 0.86)	0.008
Sex		
- Female (reference group)		
- Male	0.55 (0.35, 0.86)	0.009

Supplementary Table 6. Alternative Model 2 (n = 192): *Time to MR^4* substituted by *Previous TKI failure*, *Duration of MR^4* substituted by *Duration of TKI therapy*; *Transcript Type* emerges as an independently-significant variable. Model's optimism-corrected C-index: 0.65. Model's Bayesian information criterion (BIC): 882.4 [Main model BIC: 848.5].

Variable	Multivariable analysis for pTFR (n=192)	
	HR (95% CI)	P-value
Duration of TKI therapy (years)	0.93 (0.88, 0.98)	0.008
Previous TKI Failure		
- no (reference group)		
- yes	2.56 (1.6-4.1)	<0.0001
Age at diagnosis		
- ≤40 (reference group)		
- >40	0.57 (0.37, 0.88)	0.010
Sex		
- Female (reference group)		
- Male	0.52 (0.33, 0.81)	0.008
Transcript type		
- E14a2/e14a2-e13a2 (reference group)		
- E13a2	1.57 (1.02, 2.4)	0.039

References

1. Cross NCP, White HE, Evans PAS, Hancock J, Copland M, Milojkovic D, et al. Consensus on BCR-ABL1 reporting in chronic myeloid leukaemia in the UK. *Br J Haematol.* 2018;182(6):777-88.

2. Foroni L, Wilson G, Gerrard G, Mason J, Grimwade D, White HE, et al. Guidelines for the measurement of BCR-ABL1 transcripts in chronic myeloid leukaemia. *Br J Haematol.* 2011;153(2):179-90.

3. Cross NCP, Ernst T, Branford S, Cayuela JM, Deininger M, Fabarius A, et al. European LeukemiaNet laboratory recommendations for the diagnosis and management of chronic myeloid leukemia. Leukemia. 2023;37(11):2150-2167.

4. Dunkler D, Sauerbrei, W, & Heinze, G. Global, parameterwise and joint shrinkage factor estimation. *J Stat Softw*. 2016;69:1-19.

5. Heinze G, Wallisch C, Dunkler D. Variable selection - A review and recommendations for the practicing statistician. *Biom J.* 2018;60(3):431-449.

6. Schoenfeld, D. Partial residuals for the proportional hazards regression model. *Biometrika*. 1982;69:239-241.

7. van Buuren S, Boshuizen HC, Knook DL. Multiple imputation of missing blood pressure covariates in survival analysis. *Stat Med.* 1999;18: 681–94.

8. Royston P, Altman DG. External validation of a Cox prognostic model: principles and methods. *BMC Med Res Methodol*. 2013;13:33.

9. Patel B, Kirkwood AA, Rowntree CJ, Alapi KZ, Barretta E, Clifton-Hadley L, et al. Results from UKALL60+, a phase 2 study in older patients with untreated acute lymphoblastic leukemia. Hemasphere. 2024;8(6):e88.
